# Rhizosphere Tripartite Interactions and PGPR-Mediated Metabolic Reprogramming towards ISR and Plant Priming: A Metabolomics Review

**DOI:** 10.3390/biology11030346

**Published:** 2022-02-22

**Authors:** Manamele D. Mashabela, Lizelle A. Piater, Ian A. Dubery, Fidele Tugizimana, Msizi I. Mhlongo

**Affiliations:** 1 Research Centre for Plant Metabolomics, Department of Biochemistry, University of Johannesburg, P.O. Box 524, Auckland Park, Johannesburg 2006, South Africa; ngoatomd@gmail.com (M.D.M.); lpiater@uj.ac.za (L.A.P.); idubery@uj.ac.za (I.A.D.); Fidele.Tugizimana@omnia.co.za (F.T.); 2 International Research and Development Division, Omnia Group, Ltd., Johannesburg 2021, South Africa

**Keywords:** chemical communication, induced systemic resistance, metabolomics, plant growth-promoting rhizobacteria, priming, rhizosphere, tripartite interactions

## Abstract

**Simple Summary:**

Plant growth-promoting rhizobacteria (PGPR) are rhizosphere-inhabiting eco-friendly bacteria that indirectly/directly enhance plant growth and development and are tipped as the ‘Messiah” of plant protection against environmental stresses. However, their large-scale use in agriculture is still minimal due to the limited knowledge on them. From a metabolomics perspective, we review the current knowledge on PGPR–plant chemical communication leading to plant priming/resistant induction, highlight complex tripartite chemical interactions in the rhizosphere, and show how metabolomics can help unravel these chemical communications.

**Abstract:**

Plant growth-promoting rhizobacteria (PGPR) are beneficial microorganisms colonising the rhizosphere. PGPR are involved in plant growth promotion and plant priming against biotic and abiotic stresses. Plant–microbe interactions occur through chemical communications in the rhizosphere and a tripartite interaction mechanism between plants, pathogenic microbes and plant-beneficial microbes has been defined. However, comprehensive information on the rhizosphere communications between plants and microbes, the tripartite interactions and the biochemical implications of these interactions on the plant metabolome is minimal and not yet widely available nor well understood. Furthermore, the mechanistic nature of PGPR effects on induced systemic resistance (ISR) and priming in plants at the molecular and metabolic levels is yet to be fully elucidated. As such, research investigating chemical communication in the rhizosphere is currently underway. Over the past decades, metabolomics approaches have been extensively used in describing the detailed metabolome of organisms and have allowed the understanding of metabolic reprogramming in plants due to tripartite interactions. Here, we review communication systems between plants and microorganisms in the rhizosphere that lead to plant growth stimulation and priming/induced resistance and the applications of metabolomics in understanding these complex tripartite interactions.

## 1. Introduction

Rapid global warming and the severe effects of climate variability pose considerable threats to agricultural productivity worldwide. The concerns are further exacerbated by environmental damage, pollution and (a) biotic stresses on food crops, threatening global food security [1]. Biotic stressors come in the form of diseases caused by living organisms such as viruses, bacterial and fungal pathogens and pests, while abiotic stresses include heat and drought stress, soil salinity and environmental contaminants such as heavy metal toxicity. The types of environmental stressors mentioned above cause up to 30–70% reductions in crop productivity [1,2]. Biotic and abiotic stress conditions can severely restrict plant growth, yield or survival [3]. For instance, abiotic stress, such as salinity and water deficit, has been reported to reduce seedling growth, biomass accumulation and yield in soybean [4] and maize [5], respectively, which could impact crop growth and productivity. Due to the aforementioned factors, the threat to food security is likely to worsen, with the world population expected to reach approximately 9–10 billion people by 2050 [6,7]. The pressure on the agro-economic industry to meet the growing demand has since intensified, leading to the exhaustive use of synthetic agrochemicals to reduce stress severity, enforce pathogen and pest control and increase yields and crop productivity.

Plants have evolved complex mechanisms for defence responses under various stress conditions. These strategies may involve morphological, physiological and cytological changes, as well as transcriptional gene regulation and alterations in metabolic pathways and networks [8]. Plant defence mechanisms are essential in combatting biotic stress while helping with tolerance to abiotic stressors. A plant’s response to environmental stressors depends on its underlying genetics. The activation of defence genes induces plant immunity by a cascade of signalling molecules upon the perception of stimuli. Accordingly, the production of transgenic crops through genome editing and crossbreeding practices has been the method of choice to create stress-tolerant and disease-resistant crops as crop protection and disease management strategies [1,2,9]. In parallel, agrochemicals (i.e., fertilisers, pesticides and herbicides) have been employed to aid in plant growth, development and protection as well as eradicate pathogens [10]. However, the success rates and efficiency of these applied conventional agricultural methods are declining as they have been reported to further contribute to environmental degradation [11,12].

Phytopathogens pose one of the biggest challenges in crop management due to their versatility. Pathogens can thrive pre- and post-harvest to cause extended damage to total crop yields. The growing demand for nutritional food globally has put the agro-economic industry under pressure to develop more sustainable and environmentally friendly agricultural practices [13]. The manipulation of soil microbes is one sustainable method that can be applied to improve crop production and soil health [2]. This practice is driven by the interactions between the plant roots and microorganisms, such as plant growth-promoting rhizobacteria (PGPR) within the rhizosphere.

PGPR are plant-beneficial bacteria inhabiting the micro-ecosystem referred to as the rhizosphere that positively influence plant growth, development and productivity and offer protection against biotic and abiotic stress [14,15]. According to Adeniji et al. [16], comprehensive, holistic omics approaches such as genomic, transcriptomic and proteomic studies on pathogens and beneficial microbes have been explored and ample information on plant–microbe interactions is readily available. These studies have elucidated PGPR mechanisms of action, plant response to phytopathogen virulence and the evident outcomes of PGPR–phytopathogen interactions. However, knowledge of the biochemical mechanisms of plant–microbe interactions and the real-time metabolic changes occurring in the organisms during plant–pathogen–beneficial microbe (tripartite) interactions are scarce. On the other hand, Heinemann et al. [17] suggests that metabolomics could be used to monitor phenotypic transitions and metabolic patterns in living systems in real-time. The interpretation of these metabolic changes would lead to understanding tripartite interactions, effects of PGPR on induced systemic resistance (ISR) and plant priming. Furthermore, comprehensive studies on the parameters mentioned above would ultimately give insight into new approaches and strategies for crop protection, plant breeding and growth promotion for improved productivity and yields. Metabolomics studies (discussed in Section 2 below) have been used to elucidate plant–microbe (plant–pathogen/PGPR) interactions to uncover the potential of PGPR in crop protection [18,19,20,21,22,23]. Microbial-based crop protection strategies and the introduction of new approaches to study microbial chemical ecology have recently been highlighted [23]. However, the strategies for studying tripartite interactions are minimal, and methods of studying rhizosphere interactions are not fully representative of the nature of the ecosystem. Additionally, although advances have been made in elucidating the biochemical nature of plant–microbe interactions and the implications thereof on the plant metabolome under varying environmental conditions, major improvements are required in progressing the research as discussed below.

This review highlights the recent developments in metabolomics to study rhizosphere plant–microbe interactions, including analysis of rhizosphere exometabolites and tripartite interactions and the contribution of metabolomics technology in understanding the effects of PGPR on ISR and plant priming. The review firstly introduces metabolomics as a system biology approach to studying living systems. Secondly, a brief explanation on the concept of PGPR mechanisms of action, which have been extensively reviewed [24,25,26,27], and the rhizodeposits’ selective pressure on the rhizomicrobiome is given. Furthermore, recent metabolomics techniques in studying the rhizosphere metabolome, the literature on metabolomics studies in tripartite interactions and the current advances in deciphering the complex mechanisms and metabolic reprogramming involved in PGPR-mediated ISR and plant priming are discussed. Finally, attention is given to how metabolomics can further contribute to understanding plant–microbe interactions and the applications of the technology in crop improvement and sustainable agriculture in combination with other omics technologies.

## 2. Metabolomics as a Prospect in Integrated Systems Biology

Systems biology is an integrated, interdisciplinary field of research that aims to leverage the combined contributions of chemists, biologists, mathematicians, physicists and computational scientists in unravelling the complex mechanisms occurring in biological systems [28]. Over the years, scientists exploring systems biology approaches have made advances to seamlessly integrate multiple omics fields to quantitatively and qualitatively measure and track the intricate molecular mechanisms in organisms. The combination of various omics approaches has helped to unravel the functional dynamics of cell biology in the growth, development, adaptation and survival of organisms, as well as how the organisms interact with their surrounding environment and the underlying global biological changes [28,29].

**Figure 1:**
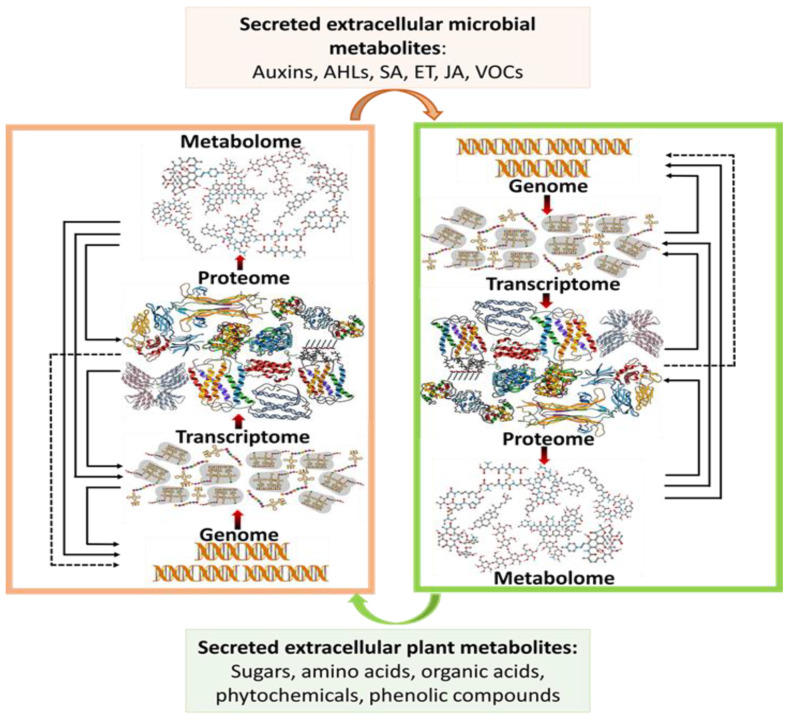
Flow of biological information across a systems biology pipeline. The figure illustrates the integrated flow of biological data through the omics system, beginning at the genome level through the metabolome. The latter creates a link between the functional phenotype and the genome, where an altered genome results in a qualitative or quantitative reprogramming of the metabolome. The intricate process allows for the regulation of cellular metabolism and maintaining homeostasis. Secondly, illustrated is the inter-organismal communication between plants and microbes. The depicted interaction creates a feedback loop that uses the metabolome of the plant and that of the microbe such as salicylic acids (SA), ethylene (ET), volatile organic compounds (VOCs) and N-acyl homoserine lactones (AHLs) as the primary mode of communication to further effect changes in the genomic level in both organisms.

Holistic approaches to biological research have made considerable progress through applied omics technologies [30,31]. The omics pipeline (Figure 1) follows the often-integrated fields of genomics, transcriptomics, proteomics and metabolomics [31]. Metabolomics is a relatively new endeavour in systems biology approaches to studying living systems and their comparative ecology. Thus, it represents a new frontier in generating a comprehensive spectrum of metabolic activities occurring in organisms. This emerging field has gained popularity due to its ability to provide detailed quantitative and qualitative analyses of a range of metabolites (small biomolecules with molecular masses of ≤1500 Da) from biological systems under specified conditions [18,19,20], and thus revealing itself as a powerful tool for answering a variety of biological questions.

**Figure 2:**
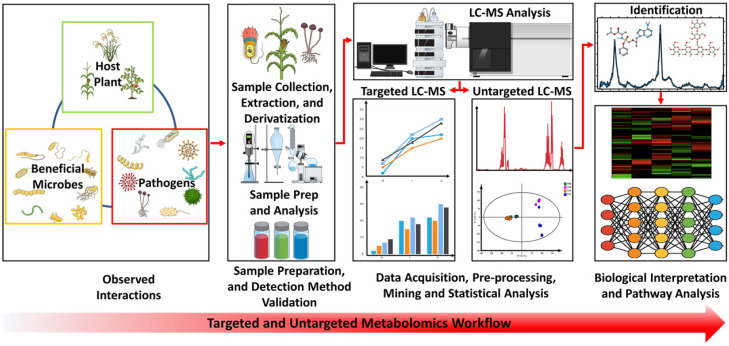
General metabolomics workflow for the analysis of plant–microbe interactions. Metabolomics studies consist of multi-step study designs which can either be targeted or untargeted. The general metabolomics workflow consists of sample preparation, data acquisition, mining and interpretation of metabolomics data. The workflow depicted above includes the general observations from the sample source in the form of tripartite interactions. Tripartite interactions (plant–phytopathogen–PGPR) result in a metabolite flux between the participating organisms, where phytopathogen metabolites assert a negative impact on both the plant and PGPR. However, such degenerative effects are countered by the beneficial defence and growth metabolites produced and secreted by the PGPR. Plants also produce and secrete metabolites as root exudates used to recruit and cater to a favourable environment for the beneficial PGPR. Such metabolomics studies have been applied to analyse the metabolic perturbations occurring in plants and PGPR when inoculated to induce heightened defence responses or promote plant growth.

The advantage of metabolomics stems from the consideration of the end products of the omics pipeline, the metabolites, thus linking the downstream phenotype and the upstream biological processes of genomics, transcriptomics and proteomics [32,33]. As such, metabolomics offers unique avenues for deciphering complex metabolic mechanisms, understanding phenotypic interpretations of genomic, transcriptomic and proteomic reprogramming and generating a platform for biomarker discovery and identifications in diagnostic studies [34,35,36]. Detailed metabolomics workflows (Figure 2) have been extensively reviewed [18,21,33]. The integration of different systems biology omics approaches provides insight into the synergistic and complementary interactions at different cellular and molecular levels. The establishment of a multi-omics system addresses gaps in our current knowledge of disease pathogenesis, and according to Diray-Arce et al. [37], this holistic approach offers opportunities for the universal understanding of a dynamic biological system. Experimentally (and methodologically), metabolomics studies are of two main approaches: targeted and untargeted approaches [20]. The former looks at detecting and quantifying pre-defined and chemically characterised metabolites from biological samples. As such, prior knowledge of the metabolites of interest is required [20,38]. In contrast, the untargeted metabolomics approach involves the global metabolic profiling and analysis of all detectable metabolites from a biological sample. This provides more information as a data-driven and hypothesis-generating aspect of metabolomics and can be very important in biomarker discovery [22,38].

Considering the complexity and diverse chemistry of metabolites in biological systems, researchers employ advanced analytical techniques that use high throughput instrumentation to provide high sensitivity and selectivity. Methods that provide reproducible results, such as mass spectrometry (MS), often hyphenated to separation techniques, including liquid or gas chromatography (L/GC), have been developed. In addition, nuclear magnetic resonance (NMR) offers the advantages of discrimination of compounds with identical masses, determination of compound structures and non-destructive sample preparation [16,22,33]. Metabolomics-based data acquisition produces large quantities of raw data that can be challenging to analyse in its multidimensional matrix form. To overcome this obstacle, metabolomics studies employ chemometrics and statistical analysis tools for data pre-processing that work synergistically to reduce the dimensionality of the data through statistical modelling. Generally, metabolite annotation, identification and biological interpretation follow [18,21,33].

## 3. Rhizosphere Metabolomics: Rhizodeposits as Agents of Rhizosphere Selective Pressure and Current Advances in Rhizosphere Exometabolite Profiling

Chemical communications between PGPR and associated plants take place in the rhizosphere. The rhizosphere is defined as the narrow region of soil immediately around the plant roots separating the bulk soil and the primary root system while also providing some of the most complex and diverse ecosystems on earth [20,39]. As such, the rhizosphere presents a dynamic, self-sustainable and heterogeneous environment in which a diverse community of microorganisms can coexist. The types of microbes generally found in the rhizosphere include bacteria (PGPR), nematodes, fungi and protozoa, with bacteria being the most abundant of these microbes [40]. The rhizosphere, in its capacity, is known to serve as the epicentre for plant–microbe and microbe–microbe interactions through info-chemical exchanges, signalling molecules and biological mechanisms. Therefore, this area provides a niche for the mutually beneficial interchange (between the plant, soil and soil rhizosphere-residing microbes) of biochemical compounds known as rhizodeposits. These compounds include proteins, lipids, carbohydrates, phytohormones and other primary and secondary metabolites (including vitamins, phenolics, steroid derivatives and antibiotics) [21,41,42].

### 3.1. Rhizosphere Plant–PGPR Communications and PGPR Mechanisms of Action

**Figure 3:**
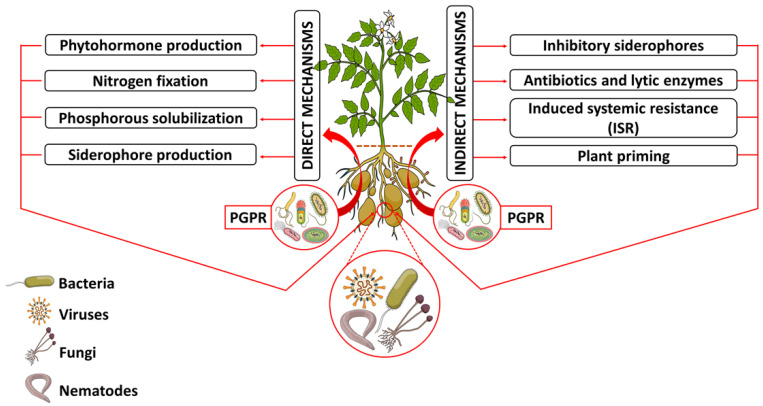
Schematic representation of the direct and indirect mechanisms of action employed by PGPR in plant growth promotion and protection. PGPR mechanisms contribute directly to plant growth by nitrogen fixation, solubilising nutrients such as phosphorous and the production and secretion of beneficial metabolites, including phytohormones and siderophores. At the same time, indirect interactions result in the protection of the plant from biotic and abiotic stress through ISR, antibiosis and plant priming.

Rhizodeposits are carbon-based, low- and high-molecular-weight compounds produced as by-products of a plant’s photosynthetic or related metabolic pathways [43]. The low-molecular-weight compounds are the most abundant in composition and offer the widest variety, thereby influencing the rhizosphere’s chemical, physical and biological processes. Thus, these compounds are responsible for shaping the microbial community of the rhizosphere [44,45]. These compounds can create a well-hydrated and nourished environment that leads to biofilm formation, which creates favourable conditions for the recruitment, growth and proliferation of PGPR [44]. PGPR have been the focus of rhizosphere plant–microbe interactions. These rhizobacteria exhibit a mutualistic relationship with the associated plants providing a range of beneficial effects during plant growth and development (Figure 3) [46].

Among the numerous roles played by PGPR, these organisms can increase plant growth rates, development and crop yields through direct mechanisms of action. PGPR are also known to apply indirect mechanisms of action to prime/sensitise plants against biotic and abiotic stresses (Figure 3) [47,48]. Hence, the recruitment of rhizobacteria into the rhizosphere has become a crucial strategy for the functioning of the ecosystem. On this note, plants hold the privilege of determining the type of bacteria favourable to colonise the rhizosphere by affecting selective pressure. The suggestion here is that plants use rhizodeposits to chemotactically attract favourable PGPR into the rhizosphere to aid in plant growth, development and defence [21,49]. This selectivity has indicated that the microbe community found in the rhizosphere can be plant species specific. It may also be dependent on the current environmental conditions or the plant developmental stages, which directly influence the exudate composition [50,51,52]. For example, Nabais et al. [53] showed the different constitutions of metabolites released in the root exudates of *Paspalum notatum* at different developmental stages of the plant. Furthermore, the study showed higher concentrations of amino acids and sugars such as arabinose at 21 days compared to 120-day-old roots. This observation further serves as an indication of age-dependent root exudation and PGPR recruitment into the rhizosphere. Moreover, it was observed by Mavrodi et al. [54] that wheat plants recruited 2,4-diacetylphloroglucinol (2,4-DAPG)-producing *Pseudomonads* under irrigated conditions. However, the same plants would recruit phenazine-producing *Pseudomonads* under dry conditions, indicating the ability to selectively recruit favourable microbes into the rhizosphere based on the current metabolic or nutritional requirements. The phenomenon described above is referred to as rhizosphere engineering [55].

PGPR can further be characterised based on the ability to colonise the rhizosphere successfully, i.e., the distinctive properties to coexist or compete with other microorganisms and efficiently interact with the plant [56,57]. The traits mentioned above determine the functional characterisation of the PGPR, which include phytostimulation, phytoremediation, bio-fertilisation and biocontrol agents (BCAs) [58]. Successful colonisation of the rhizosphere due to nutrient availability supports bacterial accumulation, growth and metabolism [59]. Among the rhizobacterial communities, the most common and widely explored genera include *Pseudomonas*, *Bacillus*, *Xanthomonas*, *Rhizobium*, *Arthrobacter*, *Azospirillum*, *Azobacter* and *Agrobacterium* [59]. Over the years, *Pseudomonas* and *Bacillus* species have been widely investigated for plant growth promotion, phytopathogen antagonism and plant priming with successful root colonisation [57]. PGPR contribute to plant growth, development and health by applying combined strategies and mechanisms to mediate and enhance already naturally occurring metabolic and physiological processes in plants [60,61]. Success can come from reduced biotic and abiotic stress on the plant, availability of essential minerals and micro-nutrients and enhanced photosynthetic capacity for increased plant biomass and crop yields [60,62], as discussed below.

**Figure 4:**
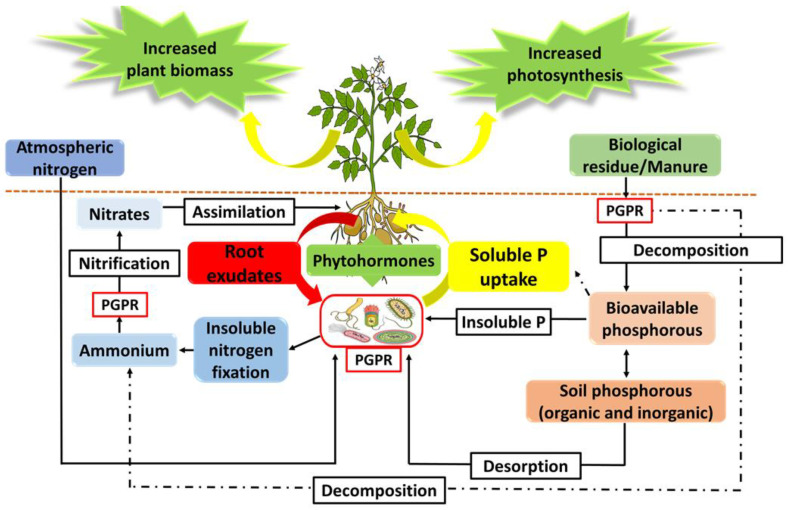
Functions of PGPR in plant growth promotion through increased biomass and photosynthetic capacity. Mineral sequestration and mobilisation by PGPR, through nitrogen fixation and phosphorous solubilisation, contributes essential minerals and nutrients to the plant resulting in improved plant biomass accumulation, photosynthesis and overall plant growth.

Research on PGPR has unravelled different mechanisms by which these organisms perform their functions, from direct plant–microbe interactions to indirect microbe–microbe interactions facilitated by the production and secretion of various substances in the rhizosphere [63]. PGPR employ two main mechanisms of action, namely a direct and indirect mechanism (Figure 3). The direct mechanism is through nutrient solubilisation and dissemination, mineral solubilisation in the form of organic and inorganic phosphorous, nitrogen fixation (Figure 4) and phytohormone and siderophore production to enhance plant growth and development [24,25]. In contrast, indirect mechanisms involve PGPR actions as BCAs, the production and secretion of volatile organic acids (VOCs), lipopeptides (LPs) and quorum sensing molecules such as N-acyl homoserine lactones (AHLs), antibiotics, siderophores and other secondary metabolites. Additionally, biotic and abiotic stress relief occurs through the induction of ISR and plant priming [22,26].

Both the plant and the rhizobacteria contribute to the diversity of the rhizosphere metabolome in an unprecedented manner due to the continuous flux of known and unknown metabolites. Moreover, the metabolite origins in the rhizosphere have not been thoroughly discriminated nor quantified. The characterisation of the rhizosphere metabolome and the determination of metabolite sources can reveal how the rhizosphere metabolite pool is impacted during plant–microbe interactions.

### 3.2. Rhizosphere Metabolomics: Current Methods and Applications

Rhizosphere metabolomics is an emerging subfield of plant metabolomics studies that looks at the comprehensive and unbiased analysis of the complete metabolome of the rhizosphere and its inhabitants. The goal is to understand the dynamics of cohabitation and the parties’ subsequent cost or physiological gains. In this context, the pathological or symbiotic relationships of rhizosphere inhabitants are scrutinised. Metabolomics studies of the rhizosphere can be very challenging due to the complex and unstable nature of the ecosystem. The continuous exudation of rhizodeposits, microbe secretions and the rhizomicrobe competition, along with the vast diversity of metabolites, can further complicate the comprehensive metabolic analyses of the region. Metabolomics studies have been popularised in recent years in addition to the more traditional or commonly applied transcriptomic and proteomic methods, more especially in plant pathological studies, in an attempt to broaden the spectrum on plant-pathogen studies from a metabolomics point of view [64]. Genomic studies and high-throughput sequencing, such as the popular *16S* rRNA sequencing, have revolutionised the characterisation of rhizosphere microbial communities. However, exploring the rhizosphere chemical diversity remains a hurdle in deciphering the core dynamics of rhizosphere communications. According to Pétriacq et al. [65], this information gap can be attributed mainly to the lack of comprehensive methods for collecting and analysing rhizosphere metabolites.

The field of metabolomics wields the power of advanced bio-analytical techniques that allow for the analysis and characterisation of complex metabolic activities in organisms. With the combination of chemometrics and bioinformatics tools, metabolomics offers the advantage of the detection and qualitative and quantitative analysis of hundreds of biochemical compounds in a single analysis [18]. Given the complexity of the rhizosphere metabolome, as previously mentioned, metabolomics provides highly selective, accurate and robust tools for untangling the complex rhizospheric plant–microbe interactions [21]. To date, many studies on root exudates have relied on hydroponics and sterile growth systems. These allow for the absolute quantification of root exudates and the collection method is conveniently simplified, thus making metabolite analysis much more straightforward. However, the techniques mentioned above do not account for the natural settings of plants under field conditions and limits access to the multi-trophic nature of the rhizosphere. Thus, the contribution of metabolites originating from the rhizomicrobes is neglected [65]. In this case, the complex biochemical diversity of non-sterile soils and rhizosphere plant–microbe signalling is not well represented. Recent studies and current advances in the applications of metabolomics in rhizosphere metabolome profiling are thus discussed.

A study by Smercina et al. [66] explored the rhizosphere chemistry of switchgrass under hydroponics systems with nitrogen (N) availability and the inoculation of N-fixing diazotroph bacteria using NMR spectroscopy analysis. Results showed improved concentrations of total metabolites in N-supplemented plants and a conclusion was drawn that diazotroph bacteria have little effect on the overall chemistry of the rhizosphere. Diazotrophs are soil-inhabiting N_2_-fixing bacteria that thrive in rhizospheric settings or bulk soils of grasses; based on this factor, an argument can be made that the unnatural environment could have reduced the efficiency of the bacteria in N_2_ fixation under the hydroponics system. From a metabolomics perspective, a similar approach was applied by Zhalnina et al. [67]. UHPLC-MS and MS/MS were used to evaluate the role of plant exudates in regulating the composition of the rhizosphere from plants grown in hydroponics tubs. Although successful plant exudate extraction was carried out, the study was performed in the absence of actual microbes; the predicted behaviour of microbes in such a setting was analysed in a separate experimental design in which extracted exudates were used as a growth medium to predict the genomic features and successional patterns of isolated bacteria in relation to the fitness of the rhizosphere.

The closest replication to the analysis of rhizosphere metabolites has been carried out by cultivating Arabidopsis plants in liquid culture media inoculated with three bacterial strains (*Pseudomonas* sp. *Root9*, *Rhizobium* sp. *Root419* and *Escherichia coli* strain *K12 MG 1655*) [68]. Analysis of extracts was performed on an HPLC hyphenated to a Q-TOF-MS analyser. Through the above study, the elements of plant–microbe interactions in real-time were achieved; however, the study reserves the argument of the profiling of exometabolites from an actual rhizosphere setting. Alternatively, plants can be grown in different soil mediums, as shown by Miller et al. [69] in a method derived from Lundberg et al. [70]. Analytes are extracted from soil samples by removing the roots with rhizosphere soil, before being placed in an extraction solvent. Shaking the root samples in the solvent is supposed to recover the root exudates; roots are then removed from the solvent and the remaining solution prepared for analysis on both GC and UHPLC-Q-TOF-MS. The method presents an effective protocol for the quantitative and qualitative analysis of rhizosphere-associated metabolites using non-targeted metabolomics profiling for belowground plant–environment interactions. However, the above method does not account for the total soil ecosystem, and the representation of the apparent plant–microbe interactions is omitted.

The knowledge gap highlighted above has led to the development of an untargeted metabolite profiling method from non-sterile rhizosphere soils. Here, Pétriacq et al. [65] described a technique for extracting polar and non-polar rhizosphere metabolites from soils hosting *Arabidopsis thaliana* and *Zea mays*. The method was designed to minimise damage to root cells and resident rhizomicrobes to avoid the potential contaminations of the targeted rhizosphere metabolites, hence recovering only those metabolites present in the rhizosphere at the time. Using UHPLC-Q-TOF mass spectrometry combined with uni- and multivariate statistical analyses, Pétriacq et al. [65] demonstrated quantitative and qualitative differences in the metabolite profiles of soils without plants and soil with plants and putatively identified discriminant rhizosphere metabolites. The study presents an advance in the comprehensive analysis of the rhizosphere metabolite profiles and unravelling the chemistry of plant–microbe interactions in complex soil environments.

Though yet to be replicated, the method by Pétriacq et al. [65] presents a straightforward protocol for profiling the rhizosphere chemistry from non-sterile soils with as close an environmental setting as the rhizosphere. Hence, the protocol brings us closer to the comprehensive analysis of rhizosphere biology and deciphering the chemistry driving the complex plant–microbe interactions in non-sterile soils. Optimisation of the method and the ones mentioned above is still required to achieve an overall representation of the total rhizosphere ecosystem; this can include testing different soils, varying extraction solutions, higher bacterial inoculum and increasing the plant biomass for maximum exudate recovery as well as increasing the number of extractions from the same soil sample. Moreover, the studies above show the prevalence of metabolomics techniques in analysing the rhizosphere metabolome and metabolomics as an emerging field for high-throughput analytical systems for studying plant–microbe interactions. In addition to rhizosphere metabolome profiling and analysis, metabolomics tools have been explored to elucidate PGPR-induced plant metabolite perturbations and tripartite interactions, as further discussed below.

## 4. Metabolomics in Understanding Microbe-Induced Plant Metabolite Perturbations and the Potential for Deciphering Plant–Microbe Tripartite Interactions: Challenges and Prospects

**Figure 5:**
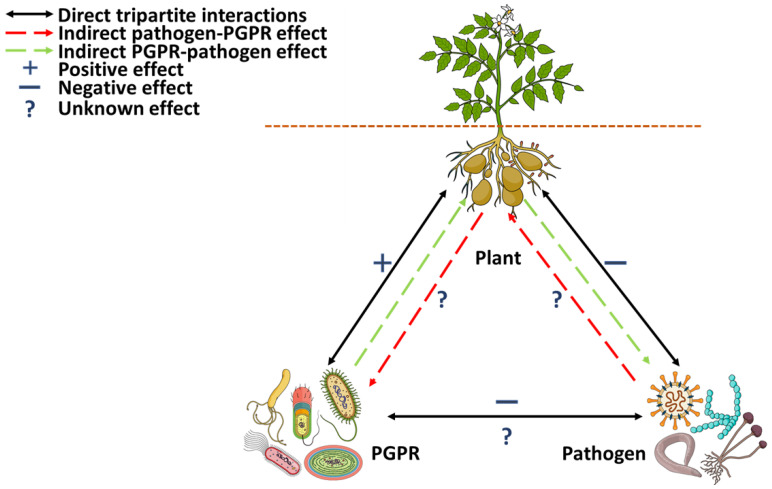
Direct and indirect tripartite interactions of plants, pathogens and PGPR. During direct tripartite interactions belowground (rhizospheric), plant–PGPR interactions result in positive effects on both parties; plant root exudates recruit and cater carbon-rich organic compounds for the proliferation of beneficial microbes (PGPR), while the microbes influence plant growth and protection. At the same time, plant–pathogen and pathogen–PGPR interactions result in negative and some unknown effects. Indirect tripartite interactions (ITI) are plant-mediated interactions between PGPR and aboveground pathogens. A negative effect on the plant by a pathogen (red dotted lines) triggers a physiological and metabolic change that causes the plant to recruit PGPR for defence. A response from the PGPR (green dotted lines) modifies the plant’s defence mechanism to improve resistance or tolerance against the foliar pathogen. Some of the underlying mechanisms of these interactions are not fully understood.

Previous studies investigating plant–microbe interactions have been extensively reviewed [21,41,71]. Many studies and literature available on plant–microbe interactions have detailed the one-way (amensalism and commensalism) or bipartite (mutualism and competition) modes of interaction [16]. Information on the three-way (tripartite) above- and belowground interactions and the role of metabolomics in tracking and discriminating metabolite origins in these interactions remains scarce (Figure 5). In an environment where organisms cohabitate, such as in tripartite interactions (plant–phytopathogen–beneficial microbe) (Figure 5), a metabolome change is expected to occur in each organism due to the continuous flux of metabolites from all participating parties. Therefore, changes in a plant’s metabolome can be affected by the metabolites produced and secreted by the beneficial microbes. Under events of infections, metabolic changes can be induced by the resident phytopathogen. Similarly, plants can cause changes in both beneficial and pathogenic microbes [16,72].

Both plant-beneficial microbes and phytopathogens modulate plant physiological processes through perturbations in the central metabolic pathways and, hence, the overall metabolome of plants [73,74]. Changes in the metabolome can also indicate alterations in the plant’s genomic, transcriptomic or proteomic profiles [36]. For example, changes due to plant–pathogen interactions may lead to metabolic pathway reprogramming to favour activation of defence genes and thus produce defence-related secondary metabolites to prevent a disease state or reduce disease progression in the plant. Similarly, a plant’s encounter with a strain of beneficial microbes (usually PGPR) can stimulate the production of primary metabolites favourable for plant growth and development. PGPR also perform antibiosis that ultimately disrupts survival mechanisms, leading to the growth and proliferation of phytopathogens. The culmination of these activities defines the concept of tripartite interactions (Figure 5).

It is essential to understand the metabolite changes taking place in plants and the resulting impacts following interactions with microbes. Knowledge of these metabolic changes can lead to significant potential in disease diagnostics, the discovery of molecular networks and the identification of significant biomarkers for plant growth and development, plant breeding for improved crop productivity and overall food security, disease resistance, stress tolerance and general plant adaptations under certain environmental conditions [75]. Previously, studies on plant–microbe interactions were focused mainly on plant–pathogen encounters. Pathogenicity and plant response to pathogen attacks were the driving force of this field of research. Invariably, the omics fields of genomics, transcriptomics and proteomics have long dictated the standards of plant pathology research. Consequently, these fields have contributed to understanding modes of pathogen infections and disease progression, for example, through genomic analysis of the genetic states of both the plant and the pathogen. Additionally, the potential modes of plant defence using transcriptomics to get insight into the manipulation and reprogramming of genes during a disease state have been revealed. Proteomics studies have generated important information on the primary mechanisms of plant–microbe interactions regarding pathogen recognition and the primary response mechanisms of plants to pathogenic invasions [76].

### 4.1. Metabolomics in the Elucidation of PGPR-Induced Plant Metabolite Perturbations

Researchers have long recognised the importance of individual metabolites such as amino acids, sugars and organic acids of the primary metabolism, or the secondary metabolites such as lipids, phytohormones, terpenes, flavonoids and alkaloids in plant–microbe interactions, more especially during disease states in plants. However, only recently have plant pathologists progressed to adopting the global analysis of these metabolites through metabolomics. The aforementioned metabolites and their functions in plant–microbe encounters reveal the complex nature of plant–microbe interactions and plant metabolism. The latter is a delicate dynamic process that is highly sensitive to external environmental stimuli. The metabolism and total metabolome pool of plants during plant–microbe interactions undergo rapid fluctuations to activate response mechanisms to the apparent stimuli [16]. The integration of plant-beneficial microbes further complicates this process to form tripartite interactions. The intricate nature of these interactions highlights the need to examine and characterise the metabolites involved. Several studies have applied metabolomics techniques to analyse the metabolite content of plant roots, root exudates and shoots to elucidate the metabolite changes resulting from PGPR inoculations. Studies on PGPR-induced plant metabolite perturbations are summarised in Table 1.

In a recent study, the analysis of *Sorghum bicolor* with UHPLC-HD-MS revealed that metabolite changes could reflect the primed state and response to fungal infection with *Colletotrichum sublineolum* following treatment with *Paenibacillus alvei* [77]. The study revealed metabolic reprogramming of 49 metabolites, including the accumulation of components of primary metabolisms such as amino acids (tyrosine and tryptophan) and resistance-related compounds of the lipid metabolism (hydroxypalmitate, epoxy-hydroxy-octadecenoate and phytosphingosine). Changes in secondary metabolism include defence-related compounds from the phenylpropanoid (e.g., luteolinidin, apigeninidin) and flavonoid (apigenin and luteolin) biosynthetic pathways that were observed along with the primary and secondary metabolism regulators such as phytohormones. The metabolic changes reported in this study pointed to the multicomponent defence response in PGPR-primed plants comprising a quicker and enhanced regulation of the primary and secondary metabolic pathways compared to the naïve (non-PGPR-primed) counterparts.

Many metabolite changes occur in plants experiencing environmental stress conditions. A recent study by Akram et al. [73] evaluated changes in the metabolome of tomato plants responding to inoculation with the halotolerant PGPR strain *B. megaterium* A12 (BMA12) under salinity stress. A UHPLC-ESI MS/MS analysis of metabolite perturbations and simultaneous phytohormone quantification revealed significant changes in abscisic acid (ABA), gibberellic acid (GA), salicylic acid (SA) and ethylene (ET) concentrations following the treatment of plants with BMA12 under saline stress. Stress-related response phytohormones such as ABA and ET were significantly increased in tomato plants under saline stress, while ET quantity was reduced in BMA12-treated plants. Reduced concentrations of stress response phytohormones in plants indicate increased stress tolerance [78].

Interestingly, the concentration of plant growth-promoting phytohormones indole-acetic acid (IAA), SA and GA were observed in BMA12-treated tomato plants, indicating the contribution of the PGPR to plant growth. Furthermore, UHPLC-ESI MS/MS analysis of the tomato plants revealed changes in the photosynthesis metabolism of the plant. Increased production of primary metabolites (sugars) such as mannose, xylose, fructose and glucose were reported in higher quantities in BMA12-treated plants than plants under saline stress in which concentrations of sugars were significantly decreased. Similar observations were made in the concentrations of amino acids, including glycine and threonine. Moreover, BMA12 symbiosis with tomato plants restored redox homeostasis and the photosynthesis system, consequently improving the growth of tomato plants against salinity stress. This study suggests that BMA12 can be applied as a potential solution in the amelioration of salinity stress.

The UHPLC-HRMS-based metabolomics was applied to investigate the physiological and metabolic changes in two chickpea genotypes and their association with drought tolerance following treatment with the PGPR *B. subtilis*, *B. thuringiensis* and *B. megaterium* in a study by Khan et al. [79]. The evaluation of significant changes in 53 out of 178 known metabolites was concomitant with physiological parameters observed through increased plant shoot and root biomass, higher accumulation of protein, sugar and phenolic compounds and enhanced leaf relative water content when comparing PGPR-treated and non-treated plants. The study showed a significant accumulation of different groups of compounds, such as amino acid: leucine; organic compounds: succinate, lactic acid, phenylpyruvate, trans-cinnamate, 2-aminophenol and malonate; sugar acid: glyceric acid; sugar: disaccharides; chemical compounds: saccharic acid and syringic acid, and ammonium compound: L-carnitine, in PGPR-treated plants as compared to control plants. The application of metabolomics tools and techniques in this study provided information that could help understand the complex PGPR-induced mechanisms occurring in plants under drought stress while highlighting PGPR contributions in plant stress tolerance. In another study, a metabolomics analysis of *Azospirillum lipoferum,* CRT1-treated maise plants showed a significant differential regulation of defence-related hydroxycinnamic acids (HCAs) and the primary metabolism through acidic intermediates of the pentose phosphate and ascorbate/aldarate pathways [80].

A combination of GC-TOF-MS and LC-ESI-MS^2^ was used by Schaker et al. [82] to elucidate metabolic perturbations in sugar cane (*Saccharum officinarum*) treated with the fungal pathogen *Sporisorium scitamineum*. According to the authors, the energy pathways in the plant, including amino acid pools, were significantly affected during the sugar cane–smut disease interaction. Here, GC-TOF-MS allowed the identification of 73 metabolites that were quantitatively altered, including xylose glycerate, raffinose and some amino acids such as tyrosine and methionine. The compounds were found to be differentially expressed with a subsequent impact on the primary cellular metabolism of the plant in the amino acid and phenylpropanoid pathways. LC-ESI-MS^2^ facilitated the identification of antifungal-related metabolites produced by the plant in response to smut disease infection. Most importantly, the study aimed to establish the link between transcriptomic changes previously reported during *Sa. Officinarum–Sp. scitamineum* interactions to subsequent metabolite alterations in the plant. The results indeed showed a correlation between transcriptomic changes and metabolite alterations in the cane plants. Taken together, the study thoroughly explores the integration of metabolomics in the systems biology arena as a complementary tool to functional genomics to explain the complex post-genomic molecular interactions and to corroborate data acquired in previous genomic analysis.

### 4.2. Applications of Metabolomics in Understanding Tripartite Plant–Microbe Interactions: Current Advances and Challenges

Studies evaluating plant responses to PGPR or phytopathogens in bipartite interactions have become prevalent in recent years with the emergence of metabolomics. However, information on the mechanistic nature of tripartite interactions is very limited. Under field conditions, PGPR do not function in isolation nor without the interference of non-beneficial microbes; hence, a metabolic interplay is expected in which alterations in the metabolomes of all organisms involved are anticipated. Changes in the metabolomes of both PGPR and pathogens can also be expected during microbe–microbe interactions; these can include synergistic PGPR–beneficial microbes or antagonistic PGPR–pathogen interactions [21], both with notable impacts on the associated plant. The metabolites released by the plant during plant–microbe interactions can also effect metabolite perturbations in the microbes; for example, MAMP-triggered immunity (MTI) induces the production of effector molecules in phytopathogens, which pathogens use to overcome MTI and elicit effector-triggered susceptibility (ETS) to advance plant infection. On the other hand, bacteria-to-bacteria communications occur through the release of signalling molecules such as VOCs and AHLs that mediate quorum sensing (QS) [21]. This form of communication can mediate long-distance interactions between beneficial microbes or effect suppressive characteristics against phytopathogenic microbes by coordinating gene expression, thus influencing virulence or stress tolerance [83]. For example, PGPR have been reported to produce antibiotics that interfere with pathogenic microbial proliferation in rhizosphere soils; these antibiotics alter the metabolic state of bacteria, thus resulting in death or stasis [84].

In the wake of newly emerging disease-causing pathogenic microbes and the development of mutated and more virulent strains of plant pathogens and growing support for the “green revolution” advocating for more sustainable disease control methods, many studies of tripartite interactions have focused on plant–PGPR–pathogen interactions. The core value of this approach was to understand the real-time plant–PGPR, plant–pathogen and PGPR–pathogen biochemical communications. Additionally, studies of the three-way relationships can help unravel the mechanisms involved in partner recognition and the crosstalk communications between plants and microbes to maintain a symbiotic relationship. Some of the earlier studies of tripartite communications explored the morphological and molecular aspects of these interactions using novel proteomic and genomic reporter systems [85,86], which investigated the changes in gene expression and differential protein production from all three participants.

Proteomic studies by Marra et al. [86] highlighted some of the earliest integration of metabolomics in the omics pipeline when MS-based protein identification was used to characterise differentially produced proteins in *Trichoderma*–plant–fungal pathogen interactions. The study applied a matrix-assisted laser desorption ionisation time-of-flight mass spectrometer (MALDI-TOF-MS) to reveal the upregulation of virulence factors such as cyclophilins in the proteomes of the pathogens *Botrytis cinerea* and *Rhizoctonia solani*. Proteins such as hydrophobins and ABC transporters were differentially regulated in the antagonistic *Trichoderma atroviride*. At the same time, the plant experienced differential regulation of pathogen- and disease-related proteins and defence genes. Since then, metabolomics approaches in the study of tripartite interactions have been scarce, which can be attributed to the difficulty in studying the complex and continuously changing micro-ecosystem under conditions that simulate the natural interactions common in agrosystems. Moreover, in recent years, metabolomics studies have taken centre stage to analyse the metabolic implication of plant–microbe interactions, mainly on the associated plant as discussed above, or defence mechanisms such as ISR. However, the potential changes in the diverse metabolomes of all three parties in the tripartite setting (on the plant and vice versa) have been neglected, especially in the pathogen. According to Adeniji et al. [16], using metabolomics to study the pathogen metabolome in this regard can offer an opportunity for the identification of phytopathogen-specific metabolic biomarkers indicative of the mechanistic nature of pathogen infection or early plant disease-specific biomarkers, which can be used to monitor the presence of a pathogen or disease progression.

The complexities of unbiased global metabolome profiling, determination of metabolite sources and tracking metabolic changes in organisms partaking in tripartite interactions have been highlighted due to the challenging community dynamics of the multitude of microbes interacting with the plant. Though a metabolomics study can offer the possibility of analysing the global metabolome in the region where the interactions occur, the lack of an established method to impartially and distinctively discriminate metabolite sources or origins is a considerable obstacle. A study by Allwood et al. [87] proposed a dual metabolomics profiling of co-cultured host plant and pathogen cells to facilitate reciprocal responses from the plant–pathogen interactions. Here, the host plant (Arabidopsis) cells and pathogen (*P. syringae* pv. *tomato* (*Pst*)) cells were co-cultured, then separated after a set period by differential filtering and centrifugation followed by endo-metabolome (fingerprinting) and exo-metabolome (footprinting) profiling using Fourier transform infrared (FT-IR) spectroscopy. The study showed distinctive responses of *Arabidopsis* cells to the pathogen. Similarly, assessment of *Pst* cells with FT-IR spectroscopy exhibited differential metabolite changes as compared to controls. The results indicate the apparent metabolite changes induced in both host plant and pathogen due to the interaction. Furthermore, to determine the identities of the metabolites responsible for the observed metabolic changes in both organisms, the analysis of the exo-metabolome (culture media alone following the removal of both plant and pathogen cells) would be carried out. Due to the dual culturing system, it should be noted that the media would be composed of metabolites from both host plant and pathogen cells. The combined (plant and pathogen) metabolite footprinting would allow for the determination of metabolite sources as the metabolites from the plant would consist mainly of sugars and antimicrobial compounds. In contrast, the pathogen cells would extrude toxins including coronatine and lipodepsipeptides [87].

A co-culturing system seems applicable to studying tripartite communications in which host plant, PGPR and pathogen cells would be cultured in the same media making up a three-way co-culture system (CCS). The question arises with metabolite attribution. Identification of some metabolites such as toxins would point to extrusion by the pathogen; similar to findings by Allwood et al. [87], most plant exudates would consist of sugars, antimicrobial compounds and other secondary defence-related metabolites specific to biotic stress conditions. Moreover, PGPR produce and secrete plant-beneficial metabolites such as phytohormones and siderophores, or antagonistic metabolites including antibiotics, lytic enzymes or pesticides [88]. This distinction of metabolites can discriminate metabolite sources in tripartite interactions, partly because plants and PGPR can still produce similar metabolites. Therefore, metabolite overlaps may thus make a clear distinction between PGPR- and plant-extruded metabolites difficult. For the challenge presented above, a bipartite CCS can be used as an alternative. Changes occurring during plant–beneficial microbe, plant–pathogen and pathogen–beneficial microbe interactions can be evaluated. Bipartite settings permit the elucidation of ex-metabolites from the organisms, giving insight into the participating organism’s metabolite exudates that potentially induce a metabolome change in the associated organism.

The core objective of studying tripartite interactions is to analyse the metabolic changes in the organisms involved; for this purpose, the CCS presents a viable analysis method. With the use of CCS combined with the most recent advanced metabolomics techniques such as UHPLC/GC coupled to time-of-flight electrospray ionisation mass spectrometry (TOF-ESI-MS), the metabolome perturbations in the relevant organisms from tripartite interactions can be elucidated. Using GC-TOF-MS and hydrophilic interaction liquid chromatography quadrupole (HILIC-Q)-TOF-MS, Saia et al. [89] studied the dynamic interactions between arbuscular mycorrhizal fungi (AMF), PGRP (*Bacillus* sp.) and wheat (*Triticum durum* Desf). The authors compared the metabolome profiles of wheat plants treated with AMF only and AMF-PGPR. It was found that AMF alone increased root colonisation, N uptake and overall plant growth. On the other hand, PGPR inoculation resulted in increased aboveground biomass, while a combined inoculation led to both higher P and aboveground biomass in the plant. The study showed the synergistic or collaborative symbiosis of the PGPR and AMF in plant growth promotion. The study by Saia et al. [89] highlights the advantages of the co-inoculation of plant-beneficial microbes to enhance plant growth promotion and development. Authors suggested that the single or co-inoculation of PGPR and AMF can contribute to improved yield, nutrient uptake and the sustainability of the agroecosystem as a valuable option for farmers. Furthermore, the co-application of PGPR can improve the biochemical responses of plants in plant–microbe interactions, thus presenting an economically viable and environmentally friendly strategy for plant growth enhancement. The review by Adeniji et al. [16] addresses the apparent knowledge gaps in the metabolomics applications for understanding complex tripartite plant–microbe interactions; the authors highlight the scarcity of information and cite the limited studies to date as a cause.

Metabolomics applications of stable isotope probing (SIP) techniques are not common in quantifying and identifying metabolites. However, SIP has been applied in the metabolite flux analysis through untargeted metabolomics to differentiate exogenous features in LC-MS to address questions such as the carbon source of microorganisms in marine sediments [90,91]. According to Chen et al. [92], heavy isotope labelling should enable the identification of the origins of specific metabolites at the side of plant–microbe interactions. For instance, plants grown in ^13^CO_2_ metabolise that available which becomes incorporated into the photosynthates ultimately released as root exudates. Using this approach, Vandenkoornhuyse et al. [93] proved root exudate uptake by Burkholderiales bacterial strains tracking ^13^C labelling of their RNA. Plants fix ^13^CO_2_ and release labelled exudates into the rhizosphere that are then consumed by the surrounding microbial community; the labelled ^13^C is further metabolised by the microbes, which are then detected in the RNA, DNA, proteins and metabolites [94]. Additionally, the assimilation of ^15^N derived from decaying matter by AMF has been reported in a study by Nuccio et al. [95]. The study applied nanometre-scale secondary ion mass spectrometry (nanoSIMS) and scanning electron microscopy (SEM) to reveal the acquisition of ^15^N by AMF and rhizobacteria that was subsequently transferred to the host plant. Potential applications of stable isotopes in deciphering root–microbe–mineral interactions and the determination of metabolite sources are thoroughly explored by Pett-Ridge et al. [94].

Our understanding of tripartite interactions, as detailed thus far, is based on direct belowground (rhizospheric) plant–PGPR–pathogen interactions (Figure 5). However, pathogenic attacks on plants are far more common on aboveground tissues, where direct interaction between the pathogen and PGPR is not feasible. In such settings as described above, the possible interaction between the pathogen and the PGPR occurs through the plant, we therefore refer to this as indirect tripartite interactions (ITI) (Figure 5). In ITI, the effect of one organism (PGPR) induces a physiological, physical or metabolic change in the intermediate (plant) which modifies the plant’s response to the third party (pathogen) that ultimately improves resistance or tolerance in the aboveground tissue (Figure 5) [96]. The systemic effects of rhizospheric plant–beneficial microbe interaction in the aboveground tissues suggest that ITI can be associated with ISR against foliar pathogens. During direct tripartite interactions in the rhizosphere, pathogens (viruses, bacteria and fungi) have a negative effect on the plant through phytotoxins and effector molecules. At the same time, pathogens are engaged in an antagonistic interaction with PGPR through active antimicrobial compounds, lytic enzymes, antibiotics and other unknown mechanisms. Additionally, the PGPR directly affects (positively) the plant through the production of plant-beneficial metabolites, while the plant caters for PGPR proliferation through carbon-rich rhizodeposits (root exudates) [97]. ITI is plant-mediated and generally occurs through an aboveground encounter with a pathogen. Pathogen infection effects a physiological or metabolic change in the plant, which causes the plant to recruit beneficial microbes (PGPR) into the rhizosphere. In turn, the PGPR induces the production of defence metabolites in the plant to restore homeostasis and promote the plant’s resistance to the pathogen [98]. Some of the mechanisms of interaction in ITI are yet to be elucidated.

According to Maldonado-Gonzalez et al. [99], the inoculation of *Arabidopsis* roots with a PICF7 strain of *P. fluorescens* induced systemic resistance and biocontrol of *Botrytis cinerea* in the leaves of the plant. This phenomenon represents a bottom-up plant-mediated ITI better exemplified by the ability of *Burkholderia phytofirmans* to migrate from the roots of a host plant to aerial parts, forming a biofilm to restrict the further proliferation of a pathogen [100]. Inversely, a top-down plant-mediated ITI occurs when the inoculation of the plant leaves with a pathogen such as *P. syringae* triggers the plant to recruit the biocontrol bacterium *B. subtilis* to colonise the roots and induce resistance against the pathogen [96].

## 5. Induced Systemic Resistance and Plant Priming: PGPR-Mediated Plant Defence and Stress Tolerance from a Metabolomics Perspective

As evidenced from the bipartite and tripartite communications discussed here, plants constantly interact with varying types of organisms, both beneficial and non-beneficial. The sessile nature of plants has constrained them to their natural environment; thus, plants have devised complex mechanisms of inter- and intra-species communications that allow for the surveillance of the immediate environment and recognising friendly or potentially harmful interactions. The plant–pathogen interactions are a classical co-evolutionary phenomenon that has been extensively studied. Over time, plants have evolved strategies and signal mechanisms to recognise stimuli from pathogen encounters, which they use to trigger and effect an immune response to mitigate or prevent pathogen attacks and disease progressions.

### PGPR-Priming/Induced Systemic Resistance

**Figure 6:**
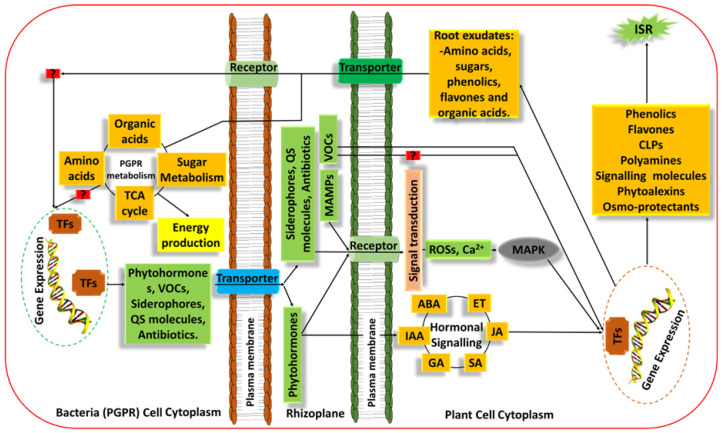
Schematic overview of the symbiotic metabolite exchange during plant–PGPR interactions in the rhizosphere. Plants secrete various organic compounds into the rhizosphere. These compounds are either taken up by the interacting organism for energy production or drive the expression of genes that lead to PGPR-derived compounds (e.g., phytohormones and volatile organic compounds) beneficial to the plant. These compounds are either perceived by the plant membrane receptors, leading to the activation of signalling cascade or diffuse through the membrane to bind to specific transcription factors to induce priming/induced systemic resistance/tolerance (ISR/T). These compounds then modulate the root exudate composition to favour the beneficial PGPR.

Plants can acquire immunity upon exposure to certain biotic or abiotic stimuli in a process that leads to the activation of enhanced (faster and more potent) inducible defence mechanisms in anticipation of subsequent antagonistic attacks. This phenomenon has been termed ‘plant priming’, also called the third layer of defence [39,101]. The detailed mechanisms by which PGPR elicit priming/ISR in plants are not yet entirely understood. However, it has been widely reported that these microbes trigger priming/ISR through the action of phytohormones as elicitors [102,103]. Additionally, metabolites such as siderophores, VOCs, QS molecules, along with lipopeptides (LPs) and MAMPs, have been implicated [104,105]. Figure 6 depicts a schematic overview of the symbiotic-metabolic exchange during plant–PGPR interactions; some of the compounds represented have been shown to play a role in the induction of ISR. Jasmonic acid (JA) and ET are the most commonly known PGPR-produced phytohormones involved in ISR elicitation and are further responsible for regulating the cascade of defence gene expression [106]. ISR induction thus requires plant responsiveness to JA and ET at the genetic and transcriptional level, a process regulated by the JA/ET signalling pathways [107]. The onset of the JA/ET signalling pathway in systemic tissue is regulated by the transcription factor (TF) MYB72. The functional JA/ET signalling pathways are further regulated by TFs MYC2 and NPR1 [108]. This pathway further activates and regulates genes such as pathogenesis-related proteins (*PR-3*), vegetative storage protein 2 (*VSP2*) and *PDF1.2* encoding defence proteins such as proteinase inhibitors and plant defensins to mediate defence responses, many of which are well characterised in *Arabidopsis* [109,110].

**Figure 7:**
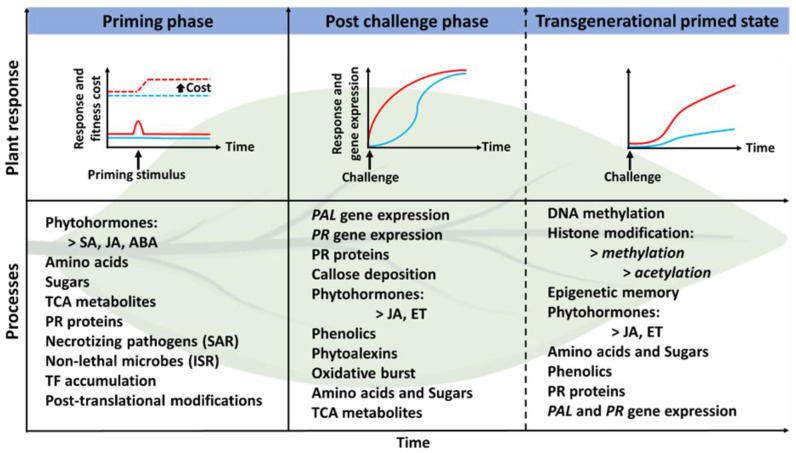
Summarised illustration of varying phases in plant priming and the induction of transgenerational memory. The scheme shows differences in plant response (solid lines) and fitness cost (dashed lines) between plants (red) and non-primed plants (blue). The perception of a priming stimulus by a plant triggers a transient defence response resulting the alteration of various primary and secondary metabolites such as amino acids, sugars, phytohormones, phenolics and proteins which comes at an initial high fitness cost as compared a non-triggered plant at the onset of priming and further maintenance of a primed state. Upon challenge by a stressor, primed plants experience low fitness cost as compared to the direct activation of defence by non-primed, which allows for an earlier, faster and more robust defence response (post-challenge phase). A transgenerational primed state is achieved when progeny plants of previously primed parents inherit epigenetic modifications regulating the state of defence priming, progeny plants are thus able to respond more robustly to a stress challenge.

In support, Beckers et al. [111] observed that priming *A. thaliana* with SA, the analogue benzothiadiazole, leads to the accumulation of mRNA transcripts along with dormant MPK3 and MPK6 proteins from sustained activation of *MPK3/6* genes. The same was observed when *Arabidopsis* leaves were challenged with the phytopathogen *P. syringae* in primed plants compared to non-primed plants. The accumulation of the MPK3/6 transcripts and proteins were implicated in sensitising the expression of the *avrRpt2* avirulent gene from *P. syringae* pv. tomato strain DC3000, leading to the sustained biosynthesis of SA in the primed plant [111,112]. As discussed earlier, SA plays a significant role in SAR induction, one of the two induced resistance mechanisms in plant priming. Further evidence of the functions of the MPKs3/6 transcripts and proteins was indicated by the reduction in priming and SA biosynthesis in Arabidopsis *mpk3/6* mutants [111]. Plant priming research dates back as far as the early 20th century when Beauverie and Ray, in 1901, discovered that the primary infection of plants with a pathogen leads to the development of an enhanced defence system that resulted in heightened defence response to secondary pathogenic infections. A primed plant is afforded the advantage of low fitness cost due to reduced resource expenditure in activating a defence response following a secondary challenge. However, successful priming and reduction in fitness cost may depend on a match between the cue and the environmental stressor to avoid misallocating resources that would ultimately negate the presumed low cost [113]. Additionally, a plant that has acquired a primed state (post-secondary challenge) from an environmental stimulus reserves an immunological memory throughout its life cycle as briefly illustrated in Figure 7. During the priming phase, a primeable organism (plant) can store information of a perceived stimulus as long- or short-term memory during the lag time between the perception of a stimulus and the encounter of a defence triggering event. The stored memory can later be retrieved to facilitate the response to a subsequent stressful environmental challenge [39]. In addition, a long-term stored memory provides opportune circumstances for epigenetic changes associated with resistance (*R*) genes, thus establishing a genetically encoded transgenerational memory for enhanced plant immunity [39,114]. The acquired immunity is ultimately passed down to the plant’s offspring through transgenerational priming, as Rasmann et al. [115] demonstrated in tomato plants responding to herbivory attack.

As discussed by Conrath et al. [116], plant priming involves alterations in the plant’s primary metabolism under investigation. As mentioned earlier, the metabolomes of co-existing organisms, either symbiotic in plant–beneficial microbe interactions or non-symbiotic, which involves plant–pathogen interactions, are expected to change as the organisms learn to adapt to their current environment. Carefully cited here as a consensus, the mechanistic nature of PGPR–plant interactions and the subsequent plant growth promotion and protection characteristic is not entirely understood. However, it has been made in the quest to elucidate these interactions and the possible exploration thereof, of PGPR in the agro-economic industry as possible sustainable substitutes for agrochemicals. According to Alberton et al. [117], proteomics and metabolomics analyses are particularly useful for elucidating these mechanisms.

The concepts of priming as discussed above describe priming mechanisms at the epigenetic, transcriptomic and proteomic levels. However, the mediation of priming mechanisms at a metabolic level remains largely unexplored, surprising given the evidence of metabolic changes recorded [77,81,118], during and post the priming phase. As such, the focus moves to the current advances in metabolomics studies to elucidate the priming mechanisms and metabolome reprogramming in plants due to PGPR-mediation of ISR and plant priming. The priming phase and the primed state of a plant are postulated by its metabolic state. Changes in the metabolic state of plants reflect the adaptation of the plant to the immediate environment, be it biotic or abiotic stress. Metabolites are the closest functional characters to the phenotype at a molecular level and are thus ubiquitous. As such, metabolic response to stress is far quicker than activities at an epigenetic or transcriptomic level, forming an essential constituent of stress response and signalling [119,120]. Therefore, post-signalling, a perturbed stress defence-related metabolic profile remains primarily due to a gradual recovery of the plant; this event causes metabolic imprints which ultimately act as stress memory storing valuable information for a plant to improve responses to future challenges [120,121].

Currently, untargeted metabolomics profiling using UHPLC-HRMS is one of the most popular analytical techniques. Untargeted metabolomics allows for the discovery and identification of metabolic biomarkers, which are very attractive to researchers due to their close association with the phenotypic characteristics of plants [81]. Mhlongo et al. [81] recently inoculated tomato plants with four different strains of PGPR (*Ps. fluorescens* N04, *Ps. koreensis* N19, *Pa. alvei* T22 and *Lysinibacillus sphaericus* T19) to study the priming effects of PGPR in tomato plants. Leaf, root and stem tissue were harvested and followed by metabolite extraction in 80% methanol. Methanol extracts were analysed on the UHPLC-MS system to acquire raw metabolomics data. Chemometric analysis revealed defence-related metabolic reprogramming through the differential, priming-related adaptations of secondary metabolites and aromatic amino acids in PGPR-treated plants compared to controls. The study showed that PGPR treatment of tomato plants leads to altered metabolite concentrations belonging to the classes of amino acids, organic acids, HCA-derivatives, flavonoids, fatty acids, benzoic acids and glycoalkaloids. The findings proposed the altered metabolites as possible signatory biomarkers of PGPR-induced plant priming. The authors suggest that the different modulation of the identified metabolites was due to the perception of external stimuli from the PGPR, which triggered the plant to modify its defence response, leading to the attainment of a pre-conditioned (primed) state and improved performance against future pathogenic attacks.

A study by Carlson et al. [77] reported on a metabolomics approach that revealed PGPR (*Pa. alvei*) treatment-related differences in sorghum seedlings’ primary and secondary metabolism following inoculation with *Fusarium pseudograminearum*. The study revealed that the differential reprogramming of metabolites in PGPR-primed plants leads to a quicker and more enhanced upregulation of metabolites involved in the defence response against *F. pseudograminearum*, showing evidence of ISR and a primed state in the plants. Furthermore, Carlson et al. [77] later investigated the effectiveness of rhizobacteria-primed plants under both biotic and abiotic stress for ISResistance, ISResilience and ISTolerance. For this study, Carlson et al. [122] exposed sorghum seedlings to a combined biotic (*F. pseudograminearum*) and abiotic (drought) stress and metabolite extracts of plants under the conditions mentioned were analysed on a UHPLC-HDMS platform for data acquisition. Similar to previous findings, results from chemometrically analysed data demonstrated differential metabolic reprogramming of PGPR-primed plants compared to their naïve counterparts (untreated control) and metabolic reprogramming was observed during the priming phase as well as in the post-challenge (primed) state. Many metabolites involved in plant defence response work synergistically to elicit a signalling cascade spanning different regulatory metabolic pathways; in this study, the treatment of *S. bicolor* with *Pa. alvei* revealed crosstalk and convergence between regulatory metabolic pathways mediated by riboflavin and glutathione metabolism under both biotic and abiotic stress conditions.

Proton ^1^H-NMR-ESI-MS was used by Abd El-Daim et al. [118] to investigate metabolic and molecular changes associated with abiotic stress mediation by *B. velezensis* 5113 in wheat. Results showed that priming of wheat with 5113 leads to metabolic reprogramming in treated seedlings and ^1^H-NMR-ESI-MS metabolite profiling revealed common stress metabolites significantly accumulated in stressed wheat, including amino acids such as L-proline and L-glutamine as well as γ-aminobutyric acid (GABA). The authors suggested that 5113 priming modulated GABA levels in stressed plants to mobilise ABA signalling to mediate stress tolerance. Furthermore, metabolomics analysis allowed for accumulated priming-related metabolites associated with metabolic pathways such as amino acid (arginine/proline), sugar and flavonoid metabolism to be identified. Another recent work in deciphering the priming mechanisms of PGPR revealed metabolic readjustments occur in both the primary and secondary metabolism of maize (*Zea mays*). The untargeted metabolomics approach by Nephali et al. [33] showed evidence of the accumulation of TCA intermediates (primary metabolism) such as fumarate and malate in PGPR-primed plants, which play a significant role in the biosynthesis of secondary defence metabolites. Additionally, changes in the secondary metabolites were observed in compounds from the classes of lipids, HCA derivatives and flavonoids. Furthermore, it was revealed that the reprogrammed metabolome of the primed plants affected metabolic pathways, including the phenylpropanoid pathway, flavonoid biosynthesis and the TCA cycle, which are involved in antioxidant regulatory mechanisms, plant cell wall reinforcements, osmoregulation and energy production.

The aforementioned studies have given insight into the complexities associated with the mechanisms of plant–microbe interactions and the processes involved in the induction of ISR and plant priming. Generally, plants are sensitised by an external stimulus in plant–microbe interactions, be it pathogenic and is prompted to respond in a defensive manner which, based on the perceived molecules (P/MAMPs), elicit the production of defence metabolites, thus leading to the upregulation or accumulation thereof in the plant. In the case of beneficial plant–microbe interactions, a defence response may still be induced. However, the plant-beneficial metabolites produced and secreted by the beneficial microbe override the plant’s defence mechanisms, leading to a symbiotic relationship. In any case, during these plant–microbe interactions, a metabolome change in the plant is expected; metabolite reprogramming on the first encounter may lead to the induction of a systemic response strategy which plants use to protect against subsequent challenges. This phenomenon is part of plant priming.

The processes involved in plant priming span a range of metabolic pathways in which associated metabolites can overlap, or the successful activation of one pathway depends on another, leading to metabolite or pathway crosstalk. Given the extent to which metabolites are involved in the regulatory pathways of plant defence, elucidation of the underlying metabolic and molecular mechanisms thereof as well as priming is challenging. In this regard, metabolomics studies have become an integral part of understanding the molecular networks and pathways involved. Furthermore, the applications of untargeted metabolomics have made possible the characterisation of phenotypes based on metabolite profiles of plants under varying environmental conditions and thus present this omics field as an essential addition to systems biology. With advancing technologies, metabolomics studies are poised to reveal new insights and more profound knowledge into the mechanistic nature of biochemical pathways involved in plant priming, ISR rhizosphere plant–microbe interactions and the future prospects of tripartite interactions.

## 6. Concluding Remarks and Perspectives

The growing global population has led to increasing demand for food production. However, the ever-increasing harmful effects of climate change, environmental pollution, the emergence of virulent pathogens and outdated conventional agricultural methods pose a considerable threat to crop and food production. Over the past decades, PGPR have emerged as a notable alternative to traditional farming practices as a means for sustainable plant growth, increased yields, enhanced plant protection for improved and sustainable food production and maintenance of food security as well as the agroeconomic industry. Plant–microbe interactions occurring in the rhizosphere have allowed for the interchange of essential metabolites between the plant and the associated PGPR. Plants deposit carbon-based organic acids as root exudates to recruit suitable PGPR, which in return produce and secrete primary and secondary metabolites essential to plant growth and defence against biotic and abiotic stress, as well as plant priming for heightened plant responses. Further advanced and more integrated plant-microbe studies have been reported along with the potential effects of PGPR as replacements for chemical fertilisers, pesticides and fungicides.

On the other hand, tripartite interactions between plants, PGPR and pathogens have indicated trends in plant metabolome and metabolic perturbations, which affect metabolic pathways and plant signalling. Highlighting the implications of pathogenic infections on the plant at a molecular and metabolic level, while PGPR have also been reported to restore the metabolome and metabolic perturbations in the plant as a form of plant growth promotion and protection from stress elements, thus restoring and preserving plant physiological processes such as photosynthesis. Furthermore, the anti-microbial effects of PGPR and their priming capabilities as ISR elicitors have been well documented, these developments have created a great interest in the abilities of PGPR as potential BCAs and agents of plant priming against biotic and abiotic stress. However, in many field conditions, the success of PGPR applications has been minimal.

Many studies would need to be accelerated in generating suitable and viable PGPR formulations for large-scale agricultural applications. Recent developments in metabolomics have allowed for detailed and comprehensive analyses of the metabolome and metabolic perturbations in plants due to environmental changes and, most notably, tripartite interactions. Identification of biomarkers and essential metabolites responsible for the observed perturbations have led to the well-documented characterisation of the effects of stressors on plants’ physiological processes. As such, the field of metabolomics has opened doors for applications in resolving the complexities in the chemical communications occurring in rhizospheric plant–microbe interactions.

## Figures and Tables

**Table 1:** Elucidated metabolome perturbations in selected plants due to plant–microbe (PGPR) interactions.

Host Plant: Source Metabolome	PGPR (Treatment) Used	Significant Metabolite Perturbations	Reference
Soybean (*Glycine max*)	*Bacillus* simplex strain Sneb545	Organic acids and amino acids	[48]
Tomato (*Solanum lycopersicum*)	*Bacillus megaterium* A12 (BMA12)	Sugars, amino acids, chlorophyll and carotenoids, antioxidants and phytohormones	[73]
Sorghum (*Sorghum bicolor*)	*Paenibacillus alvei* strain T22	Amino acids, lipids, flavonoids, phytohormones	[77]
Tomato (*Solanum lycopersicum*)	*Bacillus megaterium* A12 (BMA12)	Phytohormones	[78]
Chickpea (*Cicer arietinum*)	*Bacillus subtilis*, *Bacillus thuringiensis* and *Bacillus megaterium*	Salicylate, tryptophan, saccharic acid, glyceric acid, aminophenol, 5-oxo-L-proline, L-carnitine, trans-cinnamate, succinate and syringic acid	[79]
Maise (*Zea mays*)	*Azospirillum lipoferum* CRT1	Glucose, lactic acid, acidic intermediates of the pentose phosphate and ascorbate/aldarate pathways and defence-related hydroxycinnamic acids	[80]
Tomato (*Solanum lycopersicum*, cv. Moneymaker)	*Pseudomonas fluorescens* N04, *P. koreensis* N19, *Paenibacillus alvei* T22 and *Lysinibacillus sphaericus* T19	Hydroxycinnamic acid derivatives, benzoates, flavonoids, glycoalkaloids, fatty acids, amino acids and phytohormones	[81]
